# Preferentially Expressed Antigen of Melanoma (PRAME) and Wilms’ Tumor 1 (WT 1) Genes Expression in Childhood Acute Lymphoblastic Leukemia, Prognostic Role and Correlation with Survival

**DOI:** 10.3889/oamjms.2015.001

**Published:** 2014-12-08

**Authors:** Engy El Khateeb, Dalia Morgan

**Affiliations:** 1 *Cairo University Kasr El Aini Faculty of Medicine, Clinical Pathology, Cairo, Egypt*; 2 *Faculty of Medicine Bany Swef university, Pediatrics Department, Cairo, Egypt*

**Keywords:** ALL, PRAME, WT1, RT-PCR, cancer susceptibility, prognosis

## Abstract

**BACKGROUND:**

Acute lymphocytic leukemia (ALL) is the most common hematologic malignancy in children. In young children it is also largely curable, with more than 90% of afflicted children achieving long-term remission. PRAME (Preferentially expressed antigen of melanoma) gene belongs to Group 3 class I HLA-restricted widely expressed antigens in which genes encoding widely expressed tumor antigens have been detected in many normal tissues as well as in histologically different types of tumors with no preferential expression on a certain type of cancer. It has been found to be expressed in a variety of cancer cells as leukemia & lymphoma. PRAME monitoring can be useful for detection of minimal residual disease and subsequent relapses particularly those leukemias in which specific tumor markers are unavailable. Wilms’ tumor1 (WT1) gene was identified as a gene that plays an important role in normal kidney development and inactivation of its function was shown to result in the development of Wilms’ tumors in paediatric patients. Disruption of WT1 function has been implicated in the formation of many different tumor types.

**AIM:**

to study how PRAME & WT 1 genes expression patterns influence cancer susceptibility & prognosis.

**PATIENTS & METHODS:**

50 patients with denovo childhood acute lymphoblastic leukemia, as well as 50 age and sex matched apparently healthy volunteers were genotyped for PRAME and WT1 genes expression by reverse transcription polymerase chain reaction (RT-PCR).

**RESULTS:**

PRAME gene was expressed in 34 of the patients (68%) and WT1 gene was expressed in 26 of the patients (52%). Expression of both genes was significantly higher compared to controls (P < 0.0001). Analysis of relapse free survival among our patients revealed that patients expressing PRAME gene or WT1 gene had better relapse free survival (p value=0.02 and 0.01 respectively). Relapse free survival increased significantly among patients coexpressing PRAME and WT 1(p value =0.001).

**CONCLUSION:**

It is concluded that the expression of PRAME and WT1 genes are indicators of favorable prognosis and can be useful tools for monitoring minimal residual disease (MRD) in acute leukemia especially in patients without known genetic markers. Differential expression between acute leukemia patients and healthy volunteers suggests that the immunogenic antigens (PRAME and WT1) are potential candidates for immunotherapy in childhood acute leukemia.

## Introduction

Acute lymphoblastic leukemia (ALL) is the most common malignant disease in children younger than 15 years in Western countries [1].

The incidence of ALL decreases with age, and although it is the most common childhood cancer, the disease remains less prevalent in adolescents and adults. In pediatric studies, children over the age of 10 years display worse outcomes, but in adult registries younger patients appear to do better than older patients [2-4].

PRAME (Preferentially expressed antigen of melanoma) was first isolated as a human melanoma antigen recognized by Cytotoxic T cells (CTL). It encodes for a protein consisting of 509 amino acids and its function was unknown [5]. It has been found to be expressed in a variety of cancer cells as leukemia, lymphoma [6] and non-small-cell lung carcinoma [7], renal cell carcinoma [8], mammary carcinoma, sarcomas and head & neck tumors using semi quantitative RT-PCR [9]. PRAME monitoring can be useful for detection of minimal residual disease and subsequent relapses particularly those leukemias in which specific tumor markers are unavailable [10].

WT1 was identified as a gene that plays an important role in normal kidney development and inactivation of its function was shown to result in the development of Wilms’ tumors in paediatric patients. Using quantitative real time PCR and immunohistochemistry [11], presented data showed that WT1 is not only highly expressed in leukaemia but also in a variety of nonhaematopoetic malignancies including lung cancer, colon cancer and pancreatic carcinomas.

Constitutive expression of wild-type or mutant WT1 has been demonstrated in variety of hematologic malignancies and, particularly, in blasts of nearly all acute leukemias irrespective of lineage-specificity. Therefore, WT1 expression may be regarded as a nonspecific “panleukemic” molecular marker [12].

In normal peripheral blood (PB) and bone marrow (BM), WT1 expression is reported to be low and sometimes undetectable even by qualitative reverse transcriptase polymerase chain reaction (RT-PCR). By contrast, WT1 is highly expressed in most acute leukemias and its level of expression is associated with the presence, persistence, or reappearance of leukemic hematopoiesis [13].

Because the WT1 gene is expressed at low levels even in normal hematopoietic stem cells, most studies using qualitative or semi-quantitative analyses of WT1 transcripts have produced positive results with regard to the prediction of relapse. Early recognition of relapse at the molecular level provides a window for therapeutic intervention while the burden of disease is still relatively low [14].

The aim of this work was to study how PRAME & WT 1 genes expression patterns influence cancer susceptibility & prognosis and to be correlated with survival.

## Patients and Methods

The current study was carried out on 50 patients with denovo childhood acute lymphoblastic leukemia (ALL), in the period between June 2009 and March 2010, among cases referred to nuclear medicine and oncology unit, pediatric hospital of Kasr El Eini school of medicine, Cairo University, with follow up period of 18 months, as well as 50 age and sex matched apparently healthy volunteers. Cases were diagnosed according to WHO criteria. The diagnosis of ALL was based on morphological and phenotypic data.

Patients were 30 males and 20 females. Age ranged from 2 to 15 years. Fifty age and sex matched apparently healthy volunteers were enrolled as control group. These individuals were volunteers who had no medical history of any type of cancer or other diseases and were not related to the patients. They were 33 males and 17 females. Their ages ranged between 7and 16 years. All patients and controls were analyzed for clinical and laboratory findings, including full history taking, clinical examination, routine laboratory investigations, LDH abdominal ultrasound for detection of organomegaly and lymphadenopathy. The patients were subjected as well to cytochemical and immunophenotypic analysis to confirm diagnosis and to divide the patients into their subtypes. Written informed consent was obtained from all the participants before including them in the study.

Genotyping for PRAME and WT 1 genes expression was performed for both patients and controls. Three ml of blood were withdrawn from all the subjects included in the study in a sterile ethelenediaminetetraacetic acid (EDTA) vacutainer. Extraction of total RNA was done using High pure RNA isolation kit (Fermentas Germany catalogue number; 1828665). Patient RNA was reverse transcriped with reverse transcriptase enzyme by incubation for 30 minutes at 50°C. Following a denaturation step, the cDNA synthesis was carried out using random hexamer primers in a total volume of 25 µl. Subsequently, the cDNA was heated to inactivate the reverse transcriptase enzyme and was stored at -20°C until used for PCR amplification. PRAME and WT1 genes expression were determined by using superscript one-step RT-PCR system following the protocol from Fiedler et al., 1997 [15]. The following primer sequences were used: For PRAME gene: sense 5’-CCA TGA CAA AGA AGC GAA AA-3’ and antisense 5’-CAT CTG GCC CAG GTA AGG AG-3’, for WT-1 gene: sense: 5’ -AGAA-TACACACGCACG-GTGTCT-3’, antisense: 5’-GATGCC-GACCGTACAAGAGTC-3’ and for β actin (as an internal control): sense: 5’GGCATCGTCACCAACTGGGACGAC-3’, antisense: 5’-ATTTGCGGTGGACGATGGAGGGGC-3’.

Amplification of cDNA for PRAME gene was done for 35 cycles of: denaturation at 94°C for 1:30 minutes., annealing at 60°C for 3 minutes, extension at 68°C for 4 minutes and final extension at 68°C for 7 minutes. Amplification of cDNA for WT 1 and β actin genes: initial cycle: a precycle of 5 minutes at 94°C, 45 seconds at 60°C and 45 seconds at 68°C, PCR cycles: were 35 cycles of: Denaturation at 94°C for 1 minute, annealing at 60°C for 1 minute, extension at 68°C for 2 minutes, final extension 68°C for 7 minutes.

The most convenient method of visualizing DNA in agarose gel is by staining with the fluorescent dye ethidium bromide. The sample was considered positive when a clear, sharp, distinct band was observed at the specific molecular weight specific for PRAME (517bp), WT1 (480bp), β actin (541bp). The size of the amplified product was read with the use of a DNA marker of different molecular weights (Ladder).

### Statistics

Data were summarized and presented in the form of mean, range, percentage and standard deviation as descriptive statistics. Descriptive statistics and statistical comparison were performed using the statistical software program SPSS (version 16). Comparison was done regarding clinical data using the Chi-Square test (χ2), while for the laboratory data.

Independent T-test and ANOVA test were used. Odds ratio to access the risk related to the different gene expression. A p-value <0.05 was considered to be statistically significant.

## Results

The current study was carried out on 50 patients with childhood ALL as well as 50 age and sex matched apparently healthy volunteers (as a control group).

Patients included in our study were 30 males (60%) and 20 females (40%). Their age ranged between 2 to 15 years with a mean value of 10.2 ± 3.5. Control group were 33 males (66%) and 17 females (34%). Age ranged between 7 and 16 years with a mean of 11.2 ± 4.8. There were no statistically significant differences between the 2 groups as regard age (p value = 0.2368) or sex (p value = 0.6787). Clinical characteristics and laboratory data of patients were summarized in Table 1 and Table 2 respectively.

**Table 1:** Clinical characteristics of patients.

Clinical characteristics	Number of patients (50)	Percentage (%)
Symptoms		
-Fever	4/50	8%
-Bleeding	2/50	4%
-Neurological	15/50	30%
-Bony pains	16/50	32%
-Easy fatiguability Signs	13/50	26%
-No organomegaly	3/50	6%
-Hepatomegaly	4/50	8%
-Splenomegaly	3/50	6%
-Lymphadenopathy	40/50	80%

**Table 2:** Laboratory data of patients and controls.

Laboratory data	Patients (50)	Controls (50)	P value
Total leucocytic count/ mm³ Range Mean±SD:	2-48.5 16.7±11.9	4.5-10.5 7.8±1.8	* P < 0.0001[HS]
Hemoglobin (gm %) Range: Mean±SD:	3.5-11 7.36±2.14	11.4-14.6 13.04±1.05	* P < 0.0001[HS]
Platelets ×10³mm³ Range: Mean±SD:	4-123 56.3±32.9	155-200 173.8±13.6	* P < 0.0001[HS]

* [HS] highly significant: (P value < 0.001).

There were highly statistically significant differences between the 2 groups regarding the total leucocytic count, hemoglobin level and platelet count (p value < 0.0001).

Some additional laboratory tests and procedures were performed for the patients to diagnose ALL and to assess staging and prognostic state.

In the patient group, the serum LDH level ranged between 190-366 mg/dl with a mean of 215.6 ± 65.5, peripheral blood blast percentage ranged between 10-80 % with a mean of 37.3 ± 23.4, bone marrow aspirate blast percentage ranged between 60-98% with a mean of 80.6 ± 9.97, X rays and CT were free in 38 (76%) patients and were involved in 12 (24%) patients. CSF analysis was free in 48 (96%) patients and was involved in 2 (4%) patients.

As regard the FAB subgroups, 6 (12%) patientswere diagnosed as L1, 40 (80%) patients were diagnosed as L2, 4 (8%) patients were diagnosed as having L3. As regard the flow cytometry results, 28 (56%) patients were diagnosed as having C-ALL, while 22 (44%) patients were diagnosed as having non C-ALL.

Concerning the results of PRAME gene expression (summarized in Table 3): PRAME gene was expressed in 34 (68%) of the patients while it was only expressed in 2 (4%) of the controls.

**Table 3:** Results of PRAME and WT 1 gene expression in patients and control groups.

	Patients (n=50)	Controls (n=50)	P value
-PRAME	32/50 (64%)	2/50 (4%)	P < 0.0001[HS]*
-WT-1	28/50 (56%)	1/50 (2%)	P < 0.0001[HS]*

* [HS] highly significant: (P value < 0.001).

Concerning the results of WT1 gene expression (summarized in Table 3): WT1 gene was expressed in 26 (52%) of the patients while it was only expressed in 1 (2%) of the controls.

It was found that there were highly statistically significant differences between patient and control groups regarding PRAME and WT 1 genes expression (P < 0.0001).

PRAME and WT 1 gene expression and response to induction therapy: After chemotherapy, 29 (58%) patients achieved complete remission, 5 (10%) patients achieved only partial remission, 12 (24%) patients died, 4 (8%) patients failed to respond to induction chemotherapy.

PRAME and WT 1 gene expression and survival: Among 50 patients, 25 (50%) achieved CR, whereas 9 (18%) were resistant during the follow up period of 18 months. Death was the adverse outcome in 16 (32%) patients. The duration of relapse free survival ranged between 0 & 18 months with a mean value of 10.8 ± 5.3. At a median follow-up of 18 months, the univariate analysis revealed that the PRAME and WT 1 genes expression were significantly associated with better relapse free survival (RFS) (p value - 0.02 and 0.01, respectively). Relapse free survival increased significantly among patients coexpressing PRAME and WT 1 (p value -0.001)] (summarized in Table 4).

**Table 4:** Summarizes PRAME and WT 1 gene expression and survival.

	Relapse-free survival			

	Number of patients in CR	Median RFS (months)	18 months RFS OR (95% CI)	P value
Total	25	17.3	0.33 (0.2-0.55)	

PRAME expressed	8	17.2	0.35 (0.34-0.55)	0.02[S]*
not expressed	17	15.8	0.37 (0.35-0.56)	

WT 1 expressed	10	17.5	0.55 (0.4-0.7)	0.01[S]*
not expressed	15	16.1	0.53 (0.4-0.6)	

PRAME and WT coexpression expressed	9	17.8	0.45 (0.3-0.6)	0.001[HS]**
not expressed	16	11.1	0.53 (0.4-0.6)	

Abbreviations: CI: confidence interval ; RFS: relapse-free survival.

* Significant [S]: p value<0.05;

** [HS] highly significant: (P value < 0.001).

## Discussion

PRAME is a germinal tissue-specific gene that is also expressed at high levels in haematological malignancies and solid tumours. The physiological functions of PRAME in normal and tumour cells are unknown, although a role in the regulation of retinoic acid signalling has been proposed [16].

Our study revealed that 68% of our denovo childhood ALL patients expressed PRAME gene which was higher result in comparison to study by Steinbach et al., 2002 [17] who studied PRAME gene expression in 50 children with newly diagnosed ALL using quantitative reverse transcriptase polymerase chain reaction. Over expression of *PRAME* was found in 42% of the patients. In agreement to previous results, Greiner et al., 2006 [18] reported that PRAME gene was expressed in 42% of ALL patients. In another study, PRAME expression has been found in 15–64% of the cases with ALL, and it has been shown to correlate with t(9:22) [19]. However, PRAME was found in only 5 out of 29 (17.2%) ALL cases by Paydas et al., 2005 [20].

While PRAME is absent or expressed at very low levels in most normal tissues tested, high levels of PRAME mRNAs are encountered in malignant cells, including the vast majority of primary and metastatic melanomas (88% and 95%, respectively) [21]. Microarray and PCR studies have shown that PRAME is absent in normal haematopoietic tissues including bone marrow, CD34+ sorted bone marrow cells, unsorted peripheral blood cells and sorted B and T lymphocytes [22]. However, numerous studies have reported highly elevated levels of PRAME in both acute and chronic leukaemias and non-Hodgkin’s lymphomas [22-24].

Relapse free survival was analysed during follow up period for 18 months. We found that patients expressing PRAME gene had longer relapse free survival. Although the role of PRAME in acute leukaemia and other cancers is complex, it has promise both as a cancer biomarker and as a therapeutic target. In AML, PRAME is usually associated with a favourable response to chemotherapy and prolonged survival [17, 25]. This was initially thought to be due to its expression in leukaemias having favourable prognoses, such as AML M2 with t(8;21), AML M3 with t(15;17) and childhood B-ALL [26, 27]. However, PRAME has been reported to be an independent prognostic factor in AML M3 with t(15;17) [26] and to be associated with longer overall survival, even in karyotypes with generally poor prognosis such as deletion of the long arm of chromosome 7 and monosomy 7 [25]. In contrast, over-expression of PRAME mRNA is associated with poor prognosis in solid organ malignancies [28-30]. This raises the possibility that PRAME may have different roles in oncogenesis or tumour suppression dependent on the tumour type. Therefore, its usefulness in predicting clinical outcome in solid tumours remains unclear. However PRAME remains relevant in acute leukaemias for risk stratification, to monitor residual disease and as a potential target for immunotherapies.

WT1 gene, encodes a transcription factor involved in normal and malignant hematopoiesis [31]. In acute leukemia, WT1 mutations have been reported in 10% of patients with acute myeloid leukemia (AML). However, mutations were also observed in selected cases of acute T-lymphoblastic leukemia (T-ALL), as well as undifferentiated/biphenotypic leukemia [32]. Apart from WT1 mutations, overexpression of WT1 was found in AML patients and to a lower extent in ALL [33].

WT1 gene was expressed in 52% of our ALL patients. That was comparable to reports showing WT1expression in 44 to 86% of ALL [33, 34]. This result may depend on the higher sensitivity and specificity of the PCR. Alterations of WT1 expression (both under- or overexpression) have been described in a number of malignancies and premalignant syndromes. Remarkably, WT1 overexpression has been found in 80–90% of AML and 70–90% of ALL patients, with an even higher frequency at relapse [35-37]. The upregulation of WT1 is present in all leukemia subtypes. In AML, several authors detected lower levels of WT1 expression in more differentiated AML subtypes (M5) than in less differentiated subtypes [38], while others could not support this finding [36]. In ALL, a higher frequency of WT1 expression was found in B-ALL by one author [39] and in T-ALL by another [40]. WT1 overexpression was also detected in the cerebrospinal fluid of patients with B-ALL where it showed a strong association with disease relapse [41].

Study of relapse free survival among our patients expressing WT1 gene showed that those patients had better relapse free survival. Our findings were in contrary to other studies which identified WT1 overexpression as an independent adverse prognostic factor in acute leukemia associated with an increased risk of relapse [38, 42]. Among childhood ALL patients by Boublikova et al., 2006 [43], there was a trend toward higher WT1 expression in those who relapsed but this did not reach statistical significance. A recent study by Miglino and his collaegues, 2011 [44] on 100 patients with intermediate and poor cytogenetic risk de novo acute myeloid leukemia receiving conventional anthracycline-AraC based therapy was done. They observed that WT1 expression above 2365 was correlated also to longer event-free survival (EFS) and overall survival in the two subset of patients The positive prognostic value of high WT1 expression does not have a clear explanation; it may be implicated either with WT1 anti-oncogenic function, or with the stimulating effect of WT1 oncogene on the leukemic cellular cycle, possibly associated with an enhanced response to chemotherapy.

Our study revealed that relapse free survival increased significantly among patients coexpressing PRAME and WT genes. WT1 gene was expressed in 10 patients, 9 of them coexpressed PRAME gene (which was known to be a marker for better free survival and overall survival). This might explain why WT1 gene be considered good prognostic factor in contrary to other studies. However more studies on larger number of patients are needed for better understanding the roles of WT1 and PRAME genes in the process of leukemogenesis, their significance as a prognostic factor, minimal residual disease marker and being a possible target for immunotherapy in acute leukemia.
